# General Treatment of Fevers and Acute Internal Inflammations

**Published:** 1872-09

**Authors:** S. S. Satchwell

**Affiliations:** North Carolina


THE GEORGIA

### MEDICAL COMPANION:

A MONTHLY ADVISER.

VOL. 2. ATLANTA, GA., SEPTEMBER, 1872. No. 9.

### PART I.

### Original Communications and Special Selections.

### GENERAL TREATMENT OF FEVERS AND ACUTE INTERNAL INFLAMMATIONS.

BY S. S. SATCHWELL, M. D., OF NORTH CAROLINA.

He is the wise physician who, bringing to his daily practice a mind untrammelled by prejudice or preconceived ideas of earlier training, follows the teachings of observations, and the legitimate deductions of science. Such a practitioner, while informing himself of the views of others and the doctrines of his profession at different epochs, will at the same time carefully observe for himself. Thus armed, he will go forth to battle with disease in that spirit of independence and self-reliance which forms a material attribute of every truly successful physician. He treats his patients upon general principles, modified not only by the suggestions of each particular case, but by the surrounding circumstances and prevailing tendencies and indications. Profiting by the observations of every case, and the experience of the changing seasons and years, he heeds on the one hand neither the extreme views of blind devotees of the past, nor on the other the assumptions of the ultra advocates of the innovations and reforms of the present. But gathering as a philosopher from the past, as well as the present, avoiding those Scyllas and Charybdes of his profession that have always existed, he becomes, in truth, the genuine electric in its true sense, learned and unprejudiced while conservative and successful. Pursuing such a course of common sense and enlightened experience, he cannot fail to consult as guides the great Books of Nature and the Laws of Disease. This accustoms him to follow the methods suggested by the changing forms and types of diseases of the different seasons and years, modified by the locality, climate and varying indications of each individual case. From such stand-points arises the general treatment of every physician, who, leaving routine and pre-existent partialities and prejudices to such inferior minds as will not study and think, is governed by his own knowledge and observation as connected with the broad and established principles of his profession. Such a course may not win for him the admiration of shallow minds or the sensational applause of the fickle multitude so prone to be captivated by attractive novelties and the bold innovations of extremists. But instead thereof he will avoid the injury which such men do to science and humanity. He will, in addition, justly secure the more enviable reputation of skill and success in practice. He will gather around him the sincere friendship and lasting esteem of the learned, the sensible, and the appreciative. When ultraists and extremists have gone down in ridicule, or are forgotten in their graves, his memory will be fondly cherished, and remain to illumine the pages of Medical History.

In this section of the State, we treat disease according to indications. In one or more seasons the asthenic type is prevalent, and the tendency so strong to the typhoid form, that in acute inflammations we apply the antiphlogistic treatmant with much caution. In such years we seldom use the lancet, except in occasional instances of violent inflammation and severe congestive fevers. At other periods we find the disease more sthenic in character—the inflammatory symptoms of a higher range—cases more violent and hurry more rapidly either to resolution, if properly aided, or to a fatal end if neglected or improperly treated. Under such indications it becomes our duty to apply with a bolder hand the antiphlogistic treatment—sometimes using the lancet, more generally those mercurial purgatives, blisters, local depletion, diaphoretics, and depressants which in this locality cannot be dispensed with when our ordinary congestions and acute inflammations assume an aggravated character. So that our treatment depends upon the prevailing type and individual indications. This is the result of my observation and experience, based upon a laborious practice for the last twenty years in Eastern North Carolina. Earlier in my medical life the sthenic tide was up and my lancet was often used. For a number of years past it has become somewhat rusty, because the asthenic waves have prevailed, though it remains in my pocket case as a necessary companion. Well do I remember, as my note book shows, the high grade of many of my cases treated in earlier life, and I used the lancet freely and often with marked benefit. It was indispensable that I should open the batteries of my antiphlogistic treatment in order to save human life. But in more recent years the indications are changed—the type is lower, and I bleed more rarely and deplete less freely. I am aware that Markham, Watson and other distinguished medical men maintain the contrary theory, but the great majority of the master minds of the profession are united with the great masses of active practitioners in support of the belief that diseases change in their type. If we could discard the word type, and regard the discussion as between sthenic and asthenic forms and dispositions, much embarrassment would be prevented and extraneous matter avoided. But we must take the question as it stands: that nature is powerful, and often efficient to throw off disease, no one will deny; that the expectant system will sometimes answer, cannot be disputed. No judicious practitioner ever fails to call to his aid all the conservative strength that is possible from this source. Perhaps the truth more generally lies between the extreme advocates of the phlogistic and antiphlogistic systems. Each correct under certain circumstances, both in error under other conditions. Especially do they seem to be wrong in the uncompromising tenacity with which they hold to their peculiar views, unmodified by circumstances. However this may be, let us, I repeat, maintain ourselves free from prejudice and passion in the contests of those rival theories that have so long divided the medical world, and with the single aim of medical truth follow observation and the legitimate deductions of medical logic whithersoever they may lead.

These sentiments do not create any sympathy in me for that wholesale proscription and sweeping denunciation of the antiphlogistic system so generally indulged in by the followers of Todd, Bennett and Company. It has become too fashionable to denounce as old fogies and as enemies to true medical progress those who refuse to join the ranks of these proscriptive men. Our medical students, who go to more Northern medical colleges every year for instruction, are taught by professors who never saw a case of malarial fever, or treated a case of our high grades of internal inflammation, that it is suicidal to bleed and ignorance to give calomel, and they are taught to regard as behind this enlighted age those Southern practitioners who presume now to use in any case these antiphlogistics. I conceive it a duty, therefore, to speak out on this subject *now*, as these onslaughts upon truth and the practice and writings of a very large majority of the great leading medical minds of all ages and countries, are not conducive to the interests of medical science or humanity, so long as their heresies remain unanswered and are not exposed. I am the more disposed to discuss this question, because of the industrious efforts being made, within a recent period, to spread within our own State these dangerous doctrines of Todd, Bennet & Co. Their followers are earnestly soliciting their acceptance, by the profession of our good old State, so uniformly unwilling of her own choice to accept heresies of any kind, whether medical, political or religious. One of our own sons, a gentleman of talent and promise in his profession, a prominent member of this Society, bearing an honored name, which, by its high accomplishments and high professional lustre, has illumined the medical annals of the State, has fallen from grace, and now stands forth among us as the representative man in North Carolina of this mischievous doctrine. I need scarcely say that I allude to William A. B. Norcom, M. D., of Edenton, whose Annual Address before this Body at Warrenton, in May, 1868, has attracted much attention, and deserves to be noticed in our general allusions to the sentiments of those whom he quotes and endorses. However ungracious the task, it is due to science and the profession that such sophistry should be exposed, and such false premises and illogical deductions overthrown. Dr. Norcom's address is ingenious and well put together, but untenable and injurious upon medical practice. It is the more surprising, and to be regretted, that he has fallen into these errors, because his whole life has been passed amid disease that has presented indications, I respectfully submit, calling for different treatment than that he now advocates. Unlike those noted authors whom he quotes, scarcely one of whom knows anything practically of the nature, grade, and demands for treatment, of our Southern acute diseases, he is one of our own physicians, daily in contact, from his location, with malarious fevers and acute internal inflammations, demanding, as I insist, a more rational treatment than he applies.

The opponents of blood-letting and mercury assume, as their premises, that the progress of medical science and the revelations of truth have subverted the antiphlogistic system, and, in place thereof, have substituted alimentation, stimulants, and similar remedies. That blood-letting, mercurial purgatives and other depletants and lowering remedies, are injudicious and uncalled for, and that they prolong rather than shorten disease—increase rather than decrease mortality. Respectable medical authorities, not novel to the profession are quoted in support of these statements. Some of the very authorities quoted in support of the position taken, may be arrayed in opposition thereto in other portions of their writings. When Dr. Norcom mentions one prominent author in vindication of his views, he might have named dozens equally high in authority against himself. He fails to acknowledge that the grade of fevers and inflammations of this latitude generally produce such disturbances and indications as to demand a more vigorous treatment than do the acute diseases of the regions embraced in the fields of practice of his learned supporters in Europe and elsewhere, who wage this war against the lancet.—

There the observations of those authors are based in the main upon Hospital and Dispensary practice, when most of the cases treated have passed from the acute to the chronic stage before admission, and, of course, rendering blood-letting inadmissable. Most of their cases are asthenic in character, and different from the acute diseases of our latitude that are more phlogistic, and demand a more active treatment. "Distance," too, "lends enchantment to the view," and names arrayed here as distinguished lights in the profession and as opposed to all lowering treatment, will be found by Dr. Norcom to be more transcendental extremists, patronized but little at home and more respected abroad. They will suffer in comparison with the scores of those successful and illustrious men in all ages, who maintain adverse views, who were sensible and practical, and who contribute to adorn the brightest pages of Medical History.

Dr. Norcom sets out with the emphatic declaration that "great progress and improvement have taken place in our profession," and says that "nowhere is this more apparent than in the treatment of Acute Internal Inflammations." The improvement on this point is questionable. There is no fact to prove that the treatment of acute internal inflammations has improved. For thousands of years the antiphlogistic treatment was employed successfully by the greatest physicians—the founders of the science of medicine, to whom it owes everything, commencing with Hippocrates and running down its illumined pages to Watson, Aitkin, Wood, and others of the present day. The efficacy of the antiphlogistic plan has been attested by the experience and observations made upon millions and myriads of cases. Until the new lights can show better and more numerous authorities for the superiority of their so-called novelties (but really not) the improvement is not established.

He next says: "Foremost in importance stands the enforcement of the study of their Normal History and a revival of the *Hippocratian* doctrine of a greater reliance upon nature rather than of a perturbatory and *lowering treatment* and the substitution of a proper and sufficient alimentation for low diet—yes, we may say starvation."

The promulgation of this statement is founded upon a total misconception or misapprehension of the doctrines and practices of Hippocrates. So far from being opposed to lowering or antiphlogistic treatment, he was one of its most constant and enthusiastic supporters—in fact, one of its very founders. It is true he employed it judiciously and wisely, but on all proper occasions, especially in the treatment of acute internal inflammations, he was one of the boldest practitioners the world has ever known. This is proven by the following quotations from his works, within the reach of every physician. I quote from the translation of Dr. Francis Adams, published by the Sydenham Society of England, London, 1849:

"1st. Bleed in the acute affections, if the disease appears strong and the patients be in the vigor of life, and if they have strength.

"2. Again: Hypochondria not from retention of flatus, tension of the diaphragm, checked perspiration, with dry orthopnœ, when no pus is formed, but when these complaints are connec'ed with obstructed respiration, more especially strong pains above the diaphragm—diseases connected with a collection of humours—all these diseases do not admit of resolution if treated first by medicine, but venesection holds the first place in conducting the treatment.

"3. Again: When a person suddenly loses his speech, in connection with obstruction of the veins, if this happen without warning or any other strong cause, we ougnt to open the internal vein of the right arm and abstract blood, more or less, according to the habit and age of the patient.

"4. Again: Patients seized with Epilepsy or Apoplexy are immediately to be bled at the commencement.

"5. Again: In peri-pneumonia and pleuritic affections, bleed largely and boldly, if the pain be acute, so as to bring on *delinqum animi.*

"6. Again: When Pneumonia is at its height the case is beyond remedy, if he is not purged. In Quinsy, bleed in the arm and open the sublingual veins.

"7. Again: In Dropsy, if he labors under difficulty of breathing, if it is in the summer season, and if he is in the prime of life, blood should be abstracted from the arm.

"8. Again: The most important point of regimen to observe and be guarded about in protracted diseases, is to pay attention to the exacerbations and remissions of fever, so as to avoid the times when food should not be given, and to know when it may be administered without danger. This last season is at the greatest possible distance from the exacerbation.

"9. Again: If you think expedient to let blood, see that the bowels be previously settled, and then bleed. Conjoin abstinence, and forbid the use of wine, and complete the cure by means of a suitable regimen and wet fomentations.

"10. Again: For extreme diseases, extreme methods of cure as to restriction are most suitable. When the disease is very acute, it is attended with extremely severe symptoms in its first stage, and, therefore, an extremely attenuating diet must be used. When this is not the case, but it is allowable to give them more generous diet, we may depart in so far from the severity of regimen as the disease by its mildness is removed from the extreme. When the disease is at its height, it will then be necessary to use a more slender diet. We must retrench during paroxysms, for to exhibit food, would be injurious. And in all diseases having periodical paroxysm we must restrict during the paroxysms."

How unjust, then, to the memory of this immortal Father of Medicine, the very author of blood-letting and rigid diet, that Dr. Norcom should refer to him in support of the theory of a greater reliance on nature rather than upon a perturbatory, and lowering treatment. True, Hippocrates relied much, as all sensible men do, upon the powers of nature in the treatment of disease, but we see from the above and similar quotations that might be given from him, that, when treating severe inflammations and high fevers, he used additional means, as do judicious practitioners at this enlightened day. From these passages it will be clearly seen that Hippocrates was one of the prime originators of the lowering treatment, bleeding his patients *freely* and as largely as they would bear, and following it by rigid diet. He even forbade the use of barley water until the inflammation was arrested. How little support do such or any of the writings of Hippocrates give to the plan pursued by Todd, who says: "there is no necessity for having recourse to violent antiphlogistic measures in cases of pneumonia,"(Clinical Lectures Phil. Ed,, p. 271) and who in "rapidly spreading pneumonia" gave "half an ounce of brandy every hour," (page 266 ibid) and in another case of laryngitis, with pneumonia and delirium, gave *half an ounce of brandy* every *half hour.* It is true the patients recovered, but have they not recovered after the worst treatment ever invented ? How different the treatment of Hippocrates and that of Dr. Norcom, who, to a child eight years old, with a severa pneumonia, with pulse 140 and respiration 70 to the minute, gave three pints of milk, one and a half pints of rich soup, with *little* alcoholic stimulus every twenty-four hours ! Dr. Norcom's treatment is neither that of Hippocrates or Todd.

Leaving Hippocrates, we find that *all* the greater classics of antiquity advocated judicious antiphlogistic treatment in acute internal inflammations. Celsus recommended both venesection and cupping—(Edinburg Ed. 1814, pp. 60-40.) in pestilential and ardent fevers, he says, "if the strength will admit it is best to let blood." (Page 103.) In the case of a semi tertian he says: "unless there is some important reason against it, blood ought to be let in the beginning." In pleurisy he says: "Now the cure of a violent and recent pain is letting of blood." (Page 161.)—of pneumonia and its cure he says: "It is fit if the strength will admit of it to let blood." Of the disease of the liver and its cure he says, (page 1641) "in the beginning the best thing is to let blood, then the bowels must be opened—if this cannot be done otherwise by means of black hellebore. In ileus the cure is letting of blood."

If we turn to the pages of Galen we find that he is a strenuous advocate of antiphlogistics in all acute internal inflammations. Thus in his books on Therapeutics, addressed to Glaucus, he says: "We will say, then, that it is necessary to consider the age of the patient, the season, the country, the actual condition of the air, the strength of the patient, his complexion, his habits, and even the state of the disease. In fact you will ascertain from these whether it is necessary to evacuate or not, and how and when it ought to be done, for example in the diathesis in question. Thus if the determination is to the knee, the joint suddenly swelling greatly, if the whole body is redundant with blood, the patient vigorous, the disease occurring in the spring in a very temperate climate, and the subject a youth or young man, it will require an evacuation of blood from the upper parts, and to select from the veins of the fore-arm—the internal or median vein. If one of the superior parts are affected, the blood must be drawn from the inferior region." (Vol. 11, pp. 746–50.) Also an inflammation of the uterus, ovaries, spleen, liver, angina, affections of the head, &c., bleeding is enjoined, and the mode in which it is to be employed, and the locality of the operation minutely described—(vol. 2, pp. 755–6.)

If we consult Aretæus we find him endorsing the treatment advocated by Hippocrates and Galen. He recommends bleeding in apoplexy (page 259) and cupping (page 265) in tetanus moderately, abundantly in Cynache, with light diet (page 270). In pleurisy repeated bleedings (page 286) and cupping (291) are recommended. Repeated bleedings in pneumonia (page 296) in persons of plethoric habits, in ileus, in acute affections of the liver (page 335) and in fact in all acute diseases attended with fever and inflammation.

The observations of Paulus Ægineta, (Syd. Soc. Ed. 1844, London), one of the most celebrated writers after Galen, proves that blood-letting was the universal practice among the leading authorities of his and previous times. At the same time he employed it judiciously just like all men of good sense use everything—cautiously when doubtful, and withholding it when injurious. Paul of Egina says that "if the peripneumonia was the original affection, and the strength admit, we must open a vein, or if not we may cup, proportioning the evacuation of blood to the powers of the patient, but if the disease originate from the conversion of other diseases into this (secondary pneumonia) we must not have recourse to venesection, more especially if the disease be of a *chronic nature*, and if blood had been previously drawn." What admirable rules! In all ages we see that those who employed bleeding boldly, yet in many cases withheld it altogether. Indeed many things now promulgated as new more properly belong to the men and ages of the past. Among the Arabians, who treated pneumonia like the Greeks, and which Haley Abbas describes as a *hot inflammation* of the lungs, for which he recommended bleeding, cooling and diluent drinks, &c. We still find Rhazes describing a species of pneumonia which was treated with tonics, wine, &c. (Commentary Paulus Egineta vol. 1—482.

If we come down to Sydenham we find that in acute internal inflammations—pleurisy for example—" the fever and its most dangerous symptoms were best relieved by bleeding in the arm, applying blisters to the neck, and giving injections every day." Change in the character of disease was even then sometimes observed. He says: " Now though I conceive that a true and essential pleurisy, which, as shall hereafter be observed, happens indifferently in all constitutions, does in all years equally indicate repeated bleeding; yet it sometimes happens that the peculiar epidemic fever of the year, from some sudden alteration of the manifest qualities of the air, readily throws off the morbific matter upon the lungs and pleuræ, whilst the fever, notwithstanding, continues exactly the same. Wherefore, in this case, though bleeding may be used to abate the symptom when it is very violent, yet, generally speaking, little more blood ought to be taken away than is required by the fever whenever this *symptom* depends."—[Sydenhams Works, London, 1788, vol. 1—339.]

The great Cullen, whose authority was for a long time recognized throughout the whole medical world, was, as is well known, an advocate of the antiphlogistic treatment in acute internal inflammations and fevers. He says: " Nothing is more evident than that blood-letting is one of the most *powerful means* of diminishing the activity of the whole body, especially of the sanguinous system, and it must, therefore, be the most effectual means of moderating the violence of reaction in fevers. Taking this as a fact, I omit enquiring into its mode of operation, and shall only consider in what circumstances of fever it may be most properly employed. When the violence of reaction and its constant attendant, a phlogistic diathesis, are sufficiently manifestt, when thse constitute the principal part of the disease and may be expected to continue throughout the whole of it, as in the case of synocha, then blood-letting is the principal remedy, and may be even played as far as the symptoms of the disease may seem to require and the constitution of the patient will bear. It is, however, to be attended to, that a greater evacuation than is necessary may occasion a slower recovery; may render the person more liable to a relapse, or may bring on other diseases." He then states under what circumstances blood-letting is to be used in fevers and inflammations, and they are as follows:

"1st. The nature of the *prevailing epidemic.*

"2. The nature of the *remote cause.*

"3. The name and climate in which the disease occurs.

"4. The degree of phlogistic diathesis present.

"5. The age, vigor, and plethoric state of the patient.

"6. The former diseases and habits of blood-letting of the patient.

"7. The appearance of the blood drawn out.

"8. The effects of the blood-letting that may have been already practised."—[Vol. 1, pp. 58–59.]

To these names may be added those of Boœrhave, Andral, Chomel, Lænnec, Louis, Grisolle, McIntosh, Valleit, Gintral, Stokes, and the great medical philosopher, Bouillaud, whose directions to bleed freely in acute diseases I have heard from his own eloquent lips, as I followed him through his wards in the celebrated *de la charite* hospital at Paris. In fact there are but few physicians of note who cannot be quoted as authority in support of the antiphlogistic treatment in acute internal inflammation. And yet these great men are charged with being guilty of a reckless and profligate waste of human blood. They lay down the rules by which this treatment is to be guided, and these rules are as good now as they ever were. If our acute internal inflammations of the present day were attended by a hard, full pulse, headache, delirium, redness of face and ardent fever, we would bleed as much as ever; but this is not usually the case. The grade of reaction is lower; the type usually thyphoid or asthenic, and we have, therefore, adopted a more moderate treatment, just precisely as they *advised*, and even *commanded*. By no means would we underestimate the importance of improvements in physiology and pathology in the diagnosis and treatment of disease, but the employment of remedies is to be guided by *results*, and surely we arrogate to ourselves undue importance in assuming that we of to-day are *better judges* of *results* than the long list of illustrious names before us. The mass of the profession indeed began to employ bleeding with more caution long before the great lecturers said a word about it. Men of practical common sense in the backwoods, remote from the centres of learning, and who never read a medical book or journal, recognized this change of disease and demand for a change of treatment just as soon as this new party, so-called, of Todd, Bennet & Co. Men of common sense, who are not led off by the power of prejudice, or the charms of novelty, will profit by the lessons which these facts teach. Every physician, endowed with powers of common observation, who is conversant with the history of medicine, has a good share of clinical experience, and refuses to allow his mind to be confused by narrow circumscribed theories, is aware of them, and *has always been* and *always will be guided* by them. Men may fulminate against mercury, tartar emetic and blood-letting, still the judicious and discriminating practitioner will ply them with success, and snatch his patients from the jaws of death, at the same time that he guards against their improper use and bad effects. He will also seek constantly for such efficient aids and substitutes as observation, accident or experiment are constantly offering to his notice. He will learn something from the Natural History of Disease from Hahneman, of the use of cold water from Priestnitz, of the expectant method from the French, and of the capacity to endure alimentation and stimulants from Todd & Company. He will be ready to learn, but will not discard good remedies until others are proved to be better, numerous, repeated, prolonged trials are necessary before he gives up *Rules of Art* established for centuries.

Again, says Dr. Norcom: "Let us bear in mind that there are no foreign forces to be attacked, nor is there an excess of vitality but a deficiency of the powers which naturally reside in the organism. Indeed it may be that the cause of the attack, which demands an aid, is an already deficient vitality. I am every day more and more convinced that a recognition and observance of these important facts must form the basis of successful practice. Rather than being too intent upon driving out the enemy, let us busy ourselves, as Dr. Bennett says, to secure the safety of the fortress—let us try to bring the individual up to his physiological status. In a word, let us help him to restore his natural powers. This support can only be given by food. As Dr. Hewett says, nutrition is the basis of the treatment of disease and no other is possible for a rational system of medicine."

The plain English of this paragraph, and the gist of the whole address, is, that disease arises from *inanition*, is always asthenic in type, and always requires the same treatment—"*support*"—which can only be given by food. This is not only erroneous, but downright absurdity—not only unsupported by facts, but in direct antagonism to facts, and the daily observations of every correct medical observer. Let us give an instance: A man in the vigor of life, after having partaken of a full dinner and washed it down with brandy and wine, falls from his chair in a fit of apoplexy, shall we give him more food to cure him? Shall we attempt to induce him to eat another dinner and drink more wine, or relieve the oppressed brain by bleeding and purgatives, cold applications to the head, &c.?

A young man of full habit, (if these toddyists will admit a man *can* possess a habit *too full*) who has not missed a full meal for years, is suddenly seized with acute asthénic pneumonia, agonizing pain in the side, full bounding pulse, intense headache, difficult breathing, with a sense of weight and oppression in the chest, nausea and vomiting, what must we do? Give brandy and food? Can he digest it even if he can retain it upon his stomach? Can any sane man say that *nutrition* can be accomplished in the condition of that man's system? On the other hand, is it not known as positively as anything in medicine can be, that a free bleeding has relieved this condition of things, in some instances, as if by magic, more slowly but surely in others? Are not cases of this kind cut short by antiphlogistic treatment? Is not human life saved by such means? Let the great lights of medicine answer. Says Andral (Cour de Pathologie Intern, Tome p. 34—Paris, 1848.)—"For many centuries the treatment of pneumonia has been nearly the same, and it may be and has only varied in degree. Blood-letting has constituted its basis. The advantages which it here produces is much more direct than in other inflammations, for in diminishing the quantity of blood traversing the lung in a given time, it diminishes the activity of its functions—a result which must assist in the most efficacious manner in the cure of pneumonia." "Venesection is the method to which we ought principally to have recourse. This especially at the *commencement* of pneumonia, when the lung is only engorged that a copious bleeding often suffices to remove the disease. It is then a heroic means whose advantages can be rarely appreciated in hospitals, where the patient only arrives sometime after the invasion, but which, in civil practice, cannot be too highly recommended. Sometimes bleeding does not remove the disease, still it is not less happy in its results. As the blood flows, the patient feels his respiration less obstructed, expectoration becomes easier, the sputa less viscid and less rusty. Then the pneumonia resumes its course, and, if the bleeding is repeated, a new amelioration results."

Valleit sums up the use of bleeding in a few words, and as follows: "If the pulse is strong, full and hard, bleeding ought to be insisted upon; if it, on the contrary, becomes feeble, small and contracted, venesection should be denounced."

Louis, who certainly cannot be accused of any undue bias towards the antiphlogistic treatment, thus sums up his observations: 1st. That blood-letting has a happy influence in the march of pneumonia; that it shortens its duration, but that this influence is much less than is usually imagined; that the patients who are bled in the first few days recover, all other things being equal, four or five days sooner than those who are bled afterwards. 2nd. That pneumonia cannot be jugulated by means of bleeding, unless it is in the *first days* of the disease. 3rd. That tartar emetic given in *haute* dose when bleeding, appears to have no influence, and, therefore, the cases are grave—has a favorable action, and appears to diminish the mortality."

Lænnec, whose nice ear followed the increase and subsidence of pneumonia, says: "I treated, in 1824, at the clinic of the Faculty, with tartar emetic, twenty-eight cases of pneumonia, either simple or slightly complicated by slight pleuritic effusion. All the patients were cured except a cachetic septuagenary, who took but little antimony because he supported it badly, yet, nevertheless, the most of these cases were very grave."

No little of this antagonism of doctrine and practice seems to arise from the fact that the opposing parties are speaking of two different things—one seems to be writing of sthenic and the other of asthenic pneumonia—diseases as far apart as the antipodes. The reaction in the one case is vigorous—in the other weak. They require, of course, opposite methods of treatment. The disagreement, moreover, sometimes arises from the fact, too often disregarded, that in malarious districts a form of of disease is recognized of which malaria is an important element—a disease arising not so much from deficiency or redundancy of blood as from *poisoned* blood, which *deranging the forces regulating* the circulation of the part, gives rise to hyperemia and inflammation. In this disease bleeding can only be moderately employed with safety, and then in the exacerbation. Nor does it, like all other malarial diseases, bear alcoholic stimuli well, as is well known to practitioners in malarial regions, but is mainly controlled by a remedy which, though sedative in large doses, has an action *sui generis* in removing the local determinations and arresting the course of the febrile malady, of which they are the local expression.

But this new party, as they term themselves, though their doctrines are as old as Arabian medicine, are not content with advocating their treatment as applicable to the diseases of the present. They desire to cast odium upon the *past* history of therapeutics, thinking thereby to glorify themselves. According to their views, disease has never changed in the character of its reaction, and never will; that inflammation is always, and ever will be, the same—a position totally untenable as regards the past, and merely hypothetical in reference to the future. It need not excite surprise, if in a few years, they are found advocating bleeding, tartar emetic and calomel. They certainly must, if the pulse again becomes full, hard, bounding and resistant, as described by the old authors quoted: *Now it is soft, compressible, contracted and frequent.*

But in claiming that the recovery of their patients is due to nutriment, they forget that in the first stage of acute internal inflammation, nutrition is impossible. The patient loathes food, and if it is forced upon him his stomach cannot digest it, and it remains there a *foreign body*, or is rejected by vomiting. The very fact that digestion returns afterwards, is a proof of *convalescence*, not of *cure* induced by it. When healthy appetite returns, disease is fleeing, and the capacity for *nutrition* is a *consequence*, not a *cause* of the recovery of the patient. In how many cases of acute internal inflammation can you succeed, in inducing the patient to take food or even stimulants without marked aversion if not rejection? Nearly all fevers and inflammations commence with nausea and vomiting, furred tongue, anorexia, more or less epigastric tenderness, constipation or unhealthy stools; the secretions are arrested, diminished or prevented, the gastric fluids especially, thus rendering assimilation difficult or impossible. Do not these facts annihilate the theory of the stuffing and stimulating treatment? Does not the physiology of digestion combine with the enlightened experience in disease of every unprejudiced physician in announcing the falsity of the doctrines of this so-called new party? Do not physiology and pathology unite with therapeutics and medical experience everywhere in overwhelming Dr. Norcom's misapplied strength of alimentation and stimulation in acute internal inflammation? True, the period arrives under all methods of treatment, or under none at all, when the patient requires nourishment. Then, also, as a general rule, does he desire it. But I repel the imputation upon the great men of our profession in the past, that they *starved* their patients, and did not have sense enough to recognize the importance of "support," when the proper time came for it. That physician must indeed have read the past records of medicine with but little profit who has not found that all judicious men seized with avidity the first opportunity for building up their patients when the proper period came. Although bleeding, &c., may have been the main remedy, yet they prescribed it under certain wise rules—diminishing it, suspending it, and even rejecting it altogether, when circumstances of age, climate, country, locality, constitution, complications and anterior conditions were opposed to it. The change in the character of *disease bearing the same name*, is racognized by the great masters at every period in the history of medicine, and their treatment was varied according to circumstances. In epidemics of acute fever and inflammation of an asthenic character, bleeding was renounced by physicians a thousand years ago, and corresponding medication adopted. History denies the right of any one to pronounce it *new*, and repudiates those who now claim credit for originality.

The fear of bleeding has become a *phobia* of the day. As before intimated, swarms of young medical graduates come forth every Spring, from Northern Medical Colleges, to settle in this Southern land, their minds filled with prejudice at their institutions against venesection, calomel, &c., and boasting of the superior remedies of alimentation and stimulation in the treatment of our Southern fevers and inflammations. It is time for the South to ask whether duty to science and to ourselves does not require a change in this respect—whether we should not cease to patronize medical heresies and to seek to turn the tide of medical pupilage to our own langishing schools, whose professors, not less able nor skillful than the ablest of the North, teach doctrines generally more in unison with the laws of Southern diseases and the experience and practice of enlightened Southern practitioners. Under the constant influence of these false teachings, and such like causes, it has so much become the fashion *not* to bleed, that cases imperatively demanding it often suffer and die from its neglect. Men do not die so easily from a small loss of blood as these terror-stricken alarmists imagine. Look how many cases of protracted typhoid fever began to improve after copious hemorrhages from the nose and bowels! Every day we see *spontaneous hemorrhage* improving the condition of patients, even those suffering from tubercular consumption. Every fall, during the prevalence of those high congestive fevers of malarial character, that are every sickly season, more or less common in these eastern counties, do

I meet with cerebral congestion relieving itself by hemorrhage from the nose, when proper depletion has been neglected in the outset, and without which depletion or spontaneous hemorrhage, death would have resulted.

In regard to the expectant method of treating disease, or what amounts to the same thing, Dr. Norcom's plan of treating acute internal inflammation, there is every reason to believe that this was the first plan adopted in the infancy of the art, or even before art existed. *Men of course were led to seek for remedies because of the results of non-treatment. If the powers of nature alone* had been found *efficient to control and cure disease*, of course no one *would have wished for more.* But it must have been because this was *not* the case; it must have been owing to the *fearful and fatal* results of disease when left to the *unaided* powers of nature that men eagerly sought for remedies. Therefore, the animal, vegetable and mineral kingdoms were diligently explored to find relief from pain and protection from death.

It is fashionable to talk about a neglect of the study of the Natural History of disease being prevalent, but from the commencement of medicine this has been the object of the greatest physicians, from Hippocrates down to Louis. What better or more examples could have been wanting than in the thousands of patients placed upon the gum-water treatment of Brouissais and the wholly do-nothing treatment of Hahnemann! And yet what rational, scientific man of the present day is content with the results of their treatment? What admirable pictures of the Natural History of diseases were given by Hippocrates, is attested by every author who has perused his works. What masterly delineations of the same were given by Celsus Aretæus, and Galen, and in our day who complains of the clinical histories of disease as given by Louis, Andral and Trousseau? In cases of pneumonia we have seen that Hippocrates first used simple fomentations before bleeding. Was not this great man able to discover the relative value of expectation and venesection? · Did it require a greater genius than his to determine such a plain matter? It was clearly because men were appalled at the view of the Natural History of Disease, and the fatal results of inaction that they were induced to look for *remedies*. Moderate bleedings were often tried and failed, when subsequently copious bleedings were attended with manifest good results. We have clear examples of this fact in the ease of Cleghorn, army surgeon and lecturer of anatomy in the University of Dublin, in his universally admired and classic treatise on the Diseases of Minorca, He says: "When these Pleurisies first became epidemic their quick Progress and uncommon mortality surprised me greatly. I attempted to cure them by bleeding once or twice a Day if the Complaint were violent, as I had always used to do in Inflammatory Fevers. But the Remissions in the mornings sometimes induced me to omit the operation, and the Cessation of the symptoms, which generally happened about the third day, made me imagine the Danger was over. So that before the Patients were blooded above Twice or three Times, the Exacerbation came on upon the fourth or fifth days, and defeated all attempts by Bleeding, Blistering, or otherwise to relieve them.

"These unforeseen Events startled me greatly, and led me again to review the Progress of the Disease, its Symptoms and Issue. I had observed that some escaped by means of Expectoration and purulent Urine without much assistance from Phlebotomy; and considering the periodical Revolution of the Fever, the quick Transition of the stitches, from one Part to another, together with the prevailing Color of the Blood as well as that of the Spitting and other Excretions, I was apprehensive that these were what Authors call bilious Pleurisies, which they alledge are exasperated by large Evacuations, particularly Duretius who exclaims with great vehemence against those Physicians who trust principally to Bleeding in the cure of these Diseases, without waiting for the natural Evacuations. These motives induced me to use the lancet with more caution, and to rely chiefly on the speedy Application of Blisters for restraining the Symptoms. But this management proved less successful than the former, and I was convinced in a short time that instead of too much, too little Blood had been taken away in the beginning, having been sometimes misled by the insidious Intervals of the Disease, at others having trusted too much to the faint Attempts which Nature made to relieve herself by Expectoration and Urine, the latter, after becoming crude on the fourth day as the Delirium advanced, though it had promised favorably on the second or third, the former frequently being checked about that Period of the Disease by the immoderate Heat of the Lungs, rendering the matter viscid, globular and not to be discharged with but the utmost difficulty. I then began to bleed more plentifully, and repeated it so as to take away thirty or forty ounces within the three first days of the Distemper; and endeavored by bathing the legs and blistering them on the third Day to prevent the fatal symptoms from coming on about the fourth or fifth, giving Nitre at the same Time liberally and Camphor in small Doses to promote the thinner Secretions. This method succeeded well in several case—Expectoration and Urine being thereby increased."

This treatment, however, did not satisfy this eminent practitioner, and he at last adopted the following, as given in his own words: "If I was called, for Example in the morning, the patient was immediately laid in a horizontal position and bled at the Arm until his pulse abated or he began to faint, neither of which commonly happened before sixteen, twenty, or twenty-four ounces were taken away. If the Symptoms continued, I ordered about the same quantity to be taken from the other Arm in the Afternoon without regarding the urine, Expectoration or Appearances of the Blood, next Morning, though there might be a great Alteration for the better, yet if there was the least room to suspect that any Obstruction remained in the Head or Breast, the Bleeding was repeated. And, by carefully weighing the Blood, I found that between forty-eight and fifty-four Ounces were frequently taken away the first twenty-four Hours of my Attendance. This sudden, copious Evacuation commonly produced a cessation of all violent Symptoms, and afforded an Opportunity to give an antiphlogistic Purge the next day. But if the Symptoms did not cease, or if the Pains and difficulty of Breathing returned the day after the Purge had been given, or if there was room to suspect from the Head-ache, Giddiness, Tingling of the Ears and disturbed Rest that the Brain was in danger of being affected, I had again immediate Recourse to Bleeding, taking away at different Times to the amount of twelve, eighteen, or twenty-four Ounces in the space of a day, either by the Lancet or Cupping glassses, or both, as occasion required; by which means the impending Storm was happily averted, and as soon as the Commotions were quelled the Purgative repeated every other Day for three times unless some of the critical Evacutions appeared with such visible good effects as rendered it unnecessary. In this manner with Sydenham that Pleurisies of the most fatal Tendency might be happily cured in the space of a few days, and with as much certainty as any Distemper whatever. And it was no less remarkable to observe how quickly the Sick recovered their usual Health and Strength notwithstanding the great loss of Blood they had sustained, while many who had been bled more sparingly, continued in a languid, infirm state for months, without being able to get rid of the Cough and Pains of the Breast.—(Third London Edition, 1768. The extract is verbatim, the initial letters of the names being printed in Capital in the old English Text, as it now continues to be in the German.)

Dr. Norcom denies (page 25 of his address) that there has been any change of type in disease. He cannot be a reliable witness in the trial of this important cause, as the change occurred before he began to practice. What physician of acute observation, and who has grown gray in medical service, does not know that Dr. Norcom is in error? Who that is conversant with the History of Medicine can deny the fact of this change? Is not an ordinary autumnal Fever less severe than formerly? Where is the respectable syphilographer who will deny that syphilis is a mild disease compared to that of former days? The same may be said of Small-Pox. Is it not well known that even Asiatic Cholera is much more fatal at particular times than at others, as proven by statistics? Does not every respectable author on the subject know that Yellow Fever is sometimes a very benignant, and at other times a very malignant disease? Of the latter, witness the epidemic at Norfolk and Portsmouth, which defied all treatment, which bafled the skill of the illustrious Warren Stone as well as others of the most distinguished physicians of New Orleans, Savannah, Mobile and Charleston, as completely as it did the efforts of the merest tyro of the profession. Does not the habitual endemic of this and of Dr. Norcom's own region assume various types and degrees of violence? Most readily will the practitioners of twenty-five years experience in all our malarial regions answer this question in the affirmative. Do we not see it in some seasons attended with a short cold stage and prompt, vigorous reaction, and in others ushered in with a cold stage of days duration, and followed by imperfect reaction? Does it not assume the intermittent, remittent and continued forms? Do we not meet in some malarial seasons more than at others with that high and alarming grade of cerebral congestion, attended with that condition of the pulse so apt to mislead and deceive the unwary and inexperienced, and cause the medical attendant perhaps to neglect the golden opportunity for rescuing and saving his patient by bold and decided treatment? That physician who lives in these eastern counties and does not admit this and such as this, will do well to commence and learn over again the alphabet of his Art, or begin it for the first time. Was not the pulse of the pleurisies and pneumonias of former days as described by the great masters of medicine hard, full and resisting? Would they have continued to bleed if the lowering treatment had been attended with such deplorable results as described by Dr. Norcom? Authors who wrote on pneumonia twenty-five or thirty years ago, describe the pulse as either hard or full and resisting and bearing venesection well. Did not Cullen, Sydenham, Bœrhave, Chomel and Andral, and hosts of other observers and writers, in ancient and modern times, have as keen perception of the *results* of treatment as Bennett, Todd and Company? To ask these questions is to answer them. Let Dr. Norcom observe as intelligently and closely and laboriously as these men, and his reputation is made. They were no idlers nor dreamers, but practical, sagacious, acute observers, whose pictures of disease will forever be esteemed as master pieces, for they were drawn from Nature.

Diseases are not units. The same disease undergoes radical changes. Change is written over the pages of creation. It is a part of the sublime system of our Heavenly Father for governing the Universe and disposing of events. The changing seasons, and all that pertains to the heavens above and the earth below, proclaim this wisely ordained and universal law of Nature. The science and art of our own profession, as attested by its records, makes no exemption to the law. It teaches the true physician not alone to look at the name of disease, or its anatomical seat, but also at the patient as influenced by age, sex, temperament, diathesis, idiosyncracy, climate, locality and seasons. He does not bleed all his patients or starve them all, or stimulate them all, but is guided by the actual condition of the patient at the time of prescribing. He varies his treatment according to circumstances, and is not overruled in his judgment by any exclusive theory or blind adherence to authority. *God, as a general rule, has given every man the capacity to judge for himself as well as to receive ideas from others, and he is an unprofitable servant who does not make use of it.*

The authorities in favor of the change of type theory of disease are almost innumerable, and their arguments are unanswerable and overwhelming. It would be a work of supererogation on an occasion like this, and an unprofitable consumption of your valuable time, to array before you the host of medical philosophers and able and successful practitioners, who bear willing testimony to its truth. Among those who have presented the subject in its clearest and most convincing light, and whose arguments are most conclusive, may be named the distinguished Dr. Aitkin, the able author of the Science and Practice of Medicine, in volume first, page one hundred and thirty-nine and following. I will, in addition, cite the testimony of a former practitioner of our own State, now a prominet physician of Baltimore, and well known to this Society as one of the able Professors of the Faculty of Medicine in the University of Maryland. I allude to Professor William T. Howard. In a controversy with another eminent medical gentleman formerly of our State also, but now a leading physician of Richmond, and one of the most distinguished Professors in the Richmond Medical College—Professor Otis F. Manson. Professor Howard, in an article published several years ago in the North Carolina Medical Journal, remarked as follows: "Since the time of Hippocrates, blood-letting has been regarded by a multitude of physicians as the *remedium magnum* in pneumonia—to be used with proper discrimination and judgment; and during all this lengthened period, observing and reflecting men have practised it largely, moderately, or refrained from it entirely, according to the status of the vital forces in each individual case. Like everything else, however, blood-letting has been used in every age to great excess; and it has often happened that profuse and preposterous expenditure of the vital fluid has driven some, who witnessed its destructive effects thus practised into the opposite extreme, of abandoning it altogether. Every one knows, that within a few years past, a *great change* has taken place in the treatment of inflammatory diseases, especially in the practice of bleeding; and that, although formerly it was the rule to bleed in such affections, it has now become the exception, and is rarely resorted to. This change in practice, admitted on all hands, has been differently accounted for by different observers. We have, first, Professor J. Hughes Bennett and his party, contending that the great revolution in treatment has resulted naturally from the great advances made in modern times in diagnosis and pathology. Secondly, The late Professor Alison and his party contending that the type of disease having changed from a sthenic to an asthenic character, the practice has very properly changed accordingly; and third, Doctors Balfour and his party contending that the change has originated from neither of these circumstances, but has been forced on all alike by the results of a successful empiricism. Although the controversy involves the treatment of *inflammation* in general, yet, practically, it has turned almost entirely on the treatment of pneumonia by blood-letting."

It is beside our purpose to discuss these questions here. "Suffice it to say that with Kennedy we believe that both animal and vegetable life is subject at times to epidemic iufluences, which at one period *raise* and at another *depress* the standard of health; pneumonia, like fever, alters its type at certain times, and that no single plan of treatment can, therefore, possibly meet the ever varying shades of disease, pneumonia among the rest; or, as Watson has expressed it, we are fully persuaded both by our own observation and the records of medicine that there are waves of time through which the sthenic and asthenic characters of disease prevail in succession; and that we are at present living amid one of its adynamic phases."—(N. C. Medical Journal, March, 1860.

Again: At the same time Professor Howard continues, as follows, after comparing the results of twenty-four cases of pneumonia treated by M. Grisolle, in eleven of which bleeding was *not* employed, and in thirteen of the number it *was* used: "It seems to me that no one can compare these two series of cases together without admitting the efficacy of blood-letting on those in which it was employed. Having so often witnessed in suitable cases the influence of bleeding in reducing the force and frequency of the pulse, in diminishing the heat of the skin and rendering it moist, relieving delirium, calming restlessness, relieving headache, lessening dyspnoea, and removing or greatly moderating the pain in pneumonia, for us to doubt its utility would be wholly to discredit the evidence of our own senses."

Dr. Norcom publishes Reform doctrines *after* and not *before* the Reformation. Why denounce with such noisy clamor the antiphlogistic treatment, and especially blood-letting, when it is so sparingly used, and I may say timidly employed by the great mass of the profession now, and has been almost abandoned by a great majority of physicians? As evidence of this change, and of a correct appreciation of this reactive stage of disease, I may refer to its general recognition by those who commenced their career years before Dr. Norcom commenced his. Well do I remember my early recognition of this important medical truth, and of my corresponding shape of treatment. Let us refer to high authority in the profession of this and of other States in corroboration of this statement.

Professor Manson, already referred to, and recognized by this Society as one of the ablest and most accomplished physicians of the whole country, makes the following statements in a communication to the Stethoscope aud Virginia Medical Gazette of February, 1851: "Called to a patient in the exacerbations of remittent fever, venesecticn is practised in every case where the *pulse* will justify it, but it is *rarely* that this is called for. In fact the *general experience of myself* and confreres is *adverse* to the *use*

of the *lancet* in this affection. The pulse, though often full and apparently tense, is generally *compressible.* Local bleeding by cups and leeches will usually be sufficient, and if there is tenderness on abdominal pressure, or other symptoms of visceral complication these should be freely applied. Should symptoms of cerebral irritation be present, a copious flow of blood will be obtained by cups to the mastoidal regions."

In an article on malarial pneumonia, published in our Transactions for the year 1857, and written for the Society by the same careful observer, he says: that "the pulse which had been very frequent and contracted in the chill becomes expanded, acquires force, and sometimes, though *rarely*, becomes full or tense. The term *compressible* applies to the usual condition of pulse and the idea entertained on its careful examination is that the heart is acting with only a seeming force, and not with real vigor." When speaking of the treatment, he remarks as follows: "If the pulse is hard, full or tense, as it is in rare and exceptional cases, or if the patient is robust or previously healthy, and possessing ordinary vigor, and the pain or dyspnœa is very intense and the character of the pulse or other symptoms do not decidedly contraindicate its employment; then a moderate quantity of blood may be taken from the arm. In this disease general blood-letting should be employed with a view *only* to *moderate* and not with an expectation to cut short the disease; nor should it be employed except in the instances referred to. In cases where this is not followed by marked relief, and in those where venesection is inadmissable, the local abstraction of blood by cups or leeches should be resorted to and may be used as freely as considerations of safety will permit. The time at which bleeding by any mode should be practised, is that period when the exacerbation has reached its acme, which is almost invariably in the afternoon or evening."

These and other extracts that might be given from other authors show how careful blood-letting was used twenty years ago, when but few, if any of us, who were then coming upon the stage of action, had ever heard of the doctrines of Bennett, Todd and Company.

As to the employment of the expectant treatment, and the use of stimulants in malarial fevers, and their complications—the endemic affections of Dr. Norcom's own immediate region—I feel authorized to warn the practitioner against the habitual application of such practice. Except as a temporary means of support after these fevers have been broken up, or as an auxiliary in sustaining the vital forces in some cases of neglected treatment, while other efforts are being made to induce the action upon the system of more curative remedies, I do not believe there can be found a single experienced and judicious practitioner who will uphold the general employment of stimulants, even in those cases in which, from the very outset, there is prostration of the vital powers. If a physician gives brandy in the cold stage of congestive fever, it will end fatally in a large majority of cases; and, as to alimentation in the cold or hot stage, that is but little less than preposterous. Rather do I agree with the lamented Dr. Drake, so justly regarded in his day as the Nestor of the profession in the Northwestern States. Guided not only by his own observation, but by that of nearly every physician of note from the Great Lakes to the Gulf of Mexico, we have his high authority against the use of stimulants in these instances. In his celebrated work on the "Diseases of the Interior Valley of North America," second series, he remarks, page 86, in treating of "internal stimulants" in "malignant Intermittent Fevers," that "almost every kind of excitant and narcotico-stimulant has been administered internally in the cold stage. In this stage of the paroxysm of malignant intermittent fever, wine, brandy, whisky, and other alcoholic drinks, have been liberally given; but the results have not been such as to commend them. They probably act upon the brain unfavorably."

I have seen patients in the protracted cold stage of remittent fever, with clay-cold skin and feeble pulse, become colder and colder under the influence of stimulants. When *reaction* followed their use have seen them die in apopletic convulsions. Were we to be guided by any *a priori* theory, deduced from physiology, in our treatment of malarial fever, we should certainly expect stimulants to be useful in those cases where the vital powers are prostrate and the pulse flagging; but every sensible practitioner is perfectly aware that they are either generally *inert* or *hazardous.* If the young men coming into practice in the malarious regions of North Carolina, depend upon stimulants and alimentation in the treatment of Priodical Fevers, they will soon be compelled, like the followers of Jack o'Lantern, to find themselves in a bog, and their patients in premature graves.

Other vulnerable points in Dr. Norcom's Address consist in his omission to make any distinction in his views as to the sthenic and asthenic states in inflammation. We look in vain for an exposition of his sentiments as to the treatment of many of our every-day acute diseases. How would he treat cholera infantum, comatose remittent fever, or Asiatic cholera? Would he bring to bear his batteries of food and brandy? How long would his patients live under such treatment? How would he treat acute gastritis, entiritis, nephritis, or cystritis? With food and brandy? Would not the grave soon claim and receive them under such management? How would he treat irritis or retinitis? With food and brandy? How long would his patient be able to grope his way out of perpetual darkness? Why should Dr. Norcom, at this late day, declaim against *wholesale* blood-letting and the *abuse* of calomel and antimony, as such treatment had been abandoned before he graduated by every correct medical thinker and accurate observer? Let him take heed, clever man as he is, that he does not allow himself to be ranked among that too numerous class of the present day, who set up men of straw for the glory of knocking them down. Is he aware that his onslaughts upon antiphlogistic remedies contain unmerited strictures upon the practice of his own honored and lamented father, Dr. James Norcom? He, too, resided in Edenton, in this State, was a gentleman of profound erudition and high accomplishments, and one of the most distinguished physicians in the whole country, as he certainly was one the most successful practitioners. I cherish a peculiar pride and gratification in the belief that at, and for many years prior to, his death, he was at the head of the profession in North Carolina. What were *his* views of disease before the rolling in upon us of the "adynamic waves of the change of type?" Listen to what he says in some " Observations on the Influenza, as it appeared at

Edenton, N. C." in a letter to Dr. Rush, of Philadelphia, published in the Philadelphia Medical Museum, in 1808, volume five, page 118. " Since I returned to North Carolina I have been engaged in business with Dr. Sawyer, and have had a respectable practice, and enjoyed a greater degree of success than I ever before experienced. In the Influenza, which prevailed here from the last of September until the beginning of the year, I hardly lost a patient. The disease, in its form and character, resembled very nearly that which you have described in your Medical Inquiries. It was universally inflammatory and uniformly yielded to depletion.

"There appeared to me to be in the course of the season a greater proportion of cases with pulmonary determination than is common, and some of the most inflammatory I ever saw. One patient I bled seven times largely in forty-eight hours, and another three times in eight hours. The event in both cases was favorable. It was my happy lot to be instrumental in saving the life of an amiable woman through a series of relapses by, I am sure, not less than fifty bleedings. Bleeding, I found, in all cases of violence, an antidote to the disease, and in milder cases less direct and less active evacuants never failed to cure.

"The most fatal consequences of the fever, when it was not properly treated or speedily cured, were dropsy and consumption. Few of these have fallen to my share for a reason I have assigned already. In the dropsies that have occurred within my observation, my practice has been happy. The lancet, purging, nitre and sage tea, cream of tartar and mercury, have been my remedies. And in consumption I have cured and relieved more patients than I ever saw cured, according to the number I have attended. My remedies have been the lancet, opium, camphor, horehound and salivation."

Thus we introduce agaist Dr. Norcom, and in favor of the change of type theory, not alone the conclusive and overpowering testimony of his own dear father, but we could go on and pile authority upon authority, equally strong and convincing, in support of the positions we have taken in this paper. We have introduced our witnesses, not to advocate the propriety of their practice at this day—for, were they now in practice, they would make it correspond to the indications of the prevailing character of the disease—but our main object is to show how idle are the fears of those who condemn moderate and judicious venesection.

In relation to the general character of *Inflammation*, Dr. Norcom seems to differ in his views with those of stand?rd authors and the ablest writers. Assuming that it is unnecessary to diminish the amount of blood in an inflamed tissue, he asks: "Can general blood-letting diminish the amount of blood in an inflamed part?" In inflammatory action of the contents of the three great cavities of the head, chest and abdomen, it is found, according to high authority, that blood-letting not only diminishes the quantity of blood circulating in the vessels, but at the same time calms the turbulent action of the heart and arteries. It is admitted that in *external* inflammation, so liable to be diffused, the lancet should be used with great caution, lest by too free a abstraction of blood the constitutional symptoms already existing may be converted into fever of a different type or character. Blood-letting in internal inflammations not only diminishes the mass of circulating fluids, but is advantageous in the inflammation of external parts by drawing blood from the larger vessels going more immediately to, or returning from, these parts. It is here that the salutary influence of venesection is sure, and does not act, as Dr. Norcom insists, by materially weakening the force of the heart's action. A true interpreter of vital phenomena, a judicious observer, a stranger to the exactions of mere party systems, and unseduced by the subtleties of the schools, clearly understands that, when the excitability of the sanguineous system is carried to a very high degree, blood-letting not only diminishes the amount of blood, but in other respects proves highly beneficial. His assertion that during inflammation the vessels lose their contractile power and are distended with blood, and that stasis, owing to adhesiveness of the corpuscles, occurs, and is followed by exudation, will not be accepted. The vessels do not lose their contractile power from this cause, but during inflammation, the blood ceases to undergo its changes to the proper extent from arterial into venous, and the function of nutrition and secretion are to a very great extent suspended. On the inner surface of the walls of the dilated vessels colorless corpuscles are said to collect sometimes; but there are no new formations called forth by this change—they already exist in the blood, and when the velocity of the current is materially increased they mingle with the red corpuscles, and are carried along with them. It is not established that stasis is followed by exudation, for the reason that new canals are formed by globules of blood bursting through the sides of a vessel, and forcing a passage for themselves through the celular texture into another vessel. A considerable number of new canals are sometimes formed by this process of nature through which the blood continues to circulate. It is surely not rational or philosophic to apply to these varied phenomena any other hypothesis than the one of increased or diminished action. It appears also incorrect to describe vessels which performed their functions efficiently during health, as affected with *direct* debility because they are unable to perform double their usual labor with equal efficiency in disease. It cannot be denied that, unless under peculiar circumstances—forming exceptions to the general rule—the action of the vessels is at first greatly and powerfully increased; and it is only when they become clogged and over-distended by an excess of blood, and that, too, thinner, and more fluid than that which they contain in health, which being no longer able to contract, become passive; but it is not occasioned in consequence of injury to the *vaso motor* nerves of the part, as he contends. Adhesiveness of the corpuscles cannot, therefore, be correctly admittted as the cause of the distension of the vessels—thereby causing debility—since we only perceive this to occur as a *secondary* effect. Weak and relaxed vessels are themselves susceptible of increased action, and often in a much greater degree than vessels in an opposite state; for it is well known that constitutions in which the fibre is lax and delicate are generally characterized by a much higher degree of mobility and irritability, and are much more predisposed to inflammation than constitution endowed with a more firm and rigid texture of the solids. While delicate and sensitive vessels are easily roused into excessive action, they are less able to sustain it, and are, therefore, more readily overcome by the increased flow of blood, and more quickly affected with inflammation.

Nor will Dr. Norcom, in the assumption that there is no direct anastomosis between the surface vessels and the inflamed part, and therefore that cupping in inflammation is not profitable, secure the concurrence of his professional brethren. Let us see how this is. When a stimulus is applied to a living part, the first effect produced is an excitement of the sensibility of the part and a consequent degree of pain. The necessary result of this morbid excitement of the sensibility and contractability of the vessels is a more rapid flow of blood to the part, which acting as a stimulant tends still more to quicken the circulation. There is, consequently, a considerable influx of blood in all the vessels, capillaries and veins, to the amount of double the usual quantity. This is precisely the action which takes place in inflammations—the surface vessels in most cases anastomosing with those of the inflamed part. His opposition, therefore, to cupping cannot be sustained, and is founded upon an anatomical error, in part, because, while it is in some parts of the surface true, that the vessels from which the blood is withdrawn have no anatomical connection with those inflamed beneath, it is equally untrue as respects other parts. In all these cases of cupping, however, let it not be forgotten, whether there is or is not any direct anastomosis, that there is a *sympathy* existing through the great organic nervous centres, between the outer and inner surface or organ, by which any impression made upon the former is communicated to the latter. Herein consist a conservative law of useful results that the experience of every enlightened practitioner must have observed. It is recognized in the fact that, amid all the fluctuations of general bloodletting, the local abstraction by leeching and cupping has maintained its ground with more consistent uniformity. Local bleeding is an invaluable remedy under various circumstances of disease. When there is not much general excitement, but troublesome local congestion or inflammation; in other cases when general depletion has reduced the fullness of the pulse and moving forces of the blood with still a co-existent local inflammation; and again, where the reasons for and against the use of the lancet are so equal that it is difficult to determine as to its use, as in many cases of fever attended with inflammations; in these and similar circumstances we can often use cupping with the happiest results, even restoring health and saving life thereby in certain cases. No one conversant with the effects of both general and local bleeding will deny, that a certain quantity of blood taken from an inflamed part or near by, has decidedly more effect on the disease than a like quantity abstracted elsewhere.

### CALOMEL.

Not more unsparing and persistent have been the attacks of Todd, Bennett & Co., upon venesection than are their denunciations of the various forms of Mercury. If calomel deserves one-thousandth part of the anathemas which these and other extremists have so long hurled against its use, surely it should long since have been expelled from the Materia Medica. After all its ups and downs in professional appreciation, the "much abused Mercury" still maintains its strong hold upon the profession. True, it has been more sparingly used in the Southern States, within the last twenty-five or thirty years, and for the following reasons: The prevailing endemic and epidemic diseases were, before and since that time, of malarial origin, yet the powers of quinine were not so generally known as now. In the simple intermittents with a stage of complete apyrexia and in the benign forms of remittent fever, in which the remissions were decidedly manifest, quinine in small doses was administered by physicians generally; but in the violent cases in which the remissions were not evident, and in which symptoms of cerebral or gastro-enteric irritation, congestion, and inflammation, were either singly or unitedly present—in the large majority of cases quinine, except by a very few physicians, was usually withheld. In these cases, mercury was almost universally given. It is well known that the patient was usually pronounced "safe" as soon as ptyalism in the slightest degree presented itself. It was objected to the mercurial treatment that it was too slow and uncertain in the rapid and malignant cases, and was attended with the injurious consequences often of ptyalism, nevertheless, the mercurial treatment proved highly successful in the hands of experienced and judicious practitioners. The malignant cases which defied its powers were not so numerous as might be supposed, while the evil consequences of mercurialization were in many cases exaggerated, the majority of cases soon recovering from its effects entirely. Even after the introduction of the use of large doses of quinine, calomel continued to be necessary in completing the cure, and is still regarded as a most valuable adjuvant.

In pneumonia calomel is regarded as a most excellent remedy in skillful hands. In all my practice I can, with truth, say that I have seen but few cases of pneumonia die where the patient was clearly and timely brought under its influence. I have also seen its great beneficial influence in dysentery, diarrhœa, cholera infantum, and many others of our endemic diseases. The best, the ablest, the most successful practitioners of this and every other portion of the South have always regarded mercury as a useful and most important remedy in our Southern diseases generally. In syphilis, though for a time it fell into some disrepute under the opposition of prominent men, yet, it still holds its place in the therapeutics of nearly every modern Syphilographer.

Even the French, who have the greatest prejudices against it of any other people, have been compelled to return to its use in true chancre. Ricord still recommends it as the principal remedy. It is true that the use of mercury, even in moderate quantities, is sometimes attended with unpleasant and injurious consequences. It is true that the habit which too many of our farmers and others still pursue of keeping calomel in their houses by the pound, and of dealing it out to their families and themselves profusely, even sometimes by the teaspoonful, and for almost any and every complaint, is highly injurious and amounts to a deplorable evil; for the disintegration and disorganization of the blood is frequently induced by such mal-practice, the constitutions of their children are often undermined and ruined, and other bad consequences entailed. But this is the *abuse* and not the *proper use* of this potent remedy. The same can be said of nearly every other active agent in the Materia Medica. Opium, Arsenic, Strychnine, Iodine, Chloroform, etc., are sometimes not only injurious but fatal in their effects in the best of hands. But we are unable to discard them until better agents are provided. Of one fact we may be fully assured, and that is, neither food or whisky can ever supply the place of calomel or the other remedies in a larger majority of cases of diseases we are called upon to treat. When the *soi-disant* Modern Revolutionists in medicine *discard* them, and *not until then.* While I am ready, with Dr. Norcom, to concede it as "certain that improvements in pathology must follow in the wake of an advancing physiology," yet, in the face of all the discoveries and improvements that have been made in these departments, I utterly deny that the treatment of Acute Internal Inflammations can be based upon our present knowledge of them. Decry empiricism as we may, yet, the knowledge of the great value of therapeutic agents is chiefly due to experience and observation. Vaccination, quinine, opium, arsenic, iodine, mercury and a host of other remedies—*the best we have indeed*—were never, and could never have been, indicated or dreamed of by physiologists or pathologists. And they will continue to be employed by all endowed with common sense until greater experience and larger observation have given us better. With all our boasted knowledge of inflammation, yet, after all, how little do we really know of its *essential nature?* Do the microscopic phenomena, as observed in the web of the frog's foot, tell you anything of the *specific* nature of the *varieties* of inflammation, of the *causes* or *cure?* How idle then, to attempt to base the art of medicine upon Physiology or Pathological Anatomy! They are simply accessories—aids—but not the corner stones of therapeutics. Dispute as we will about the *modus operandi* of medicines, yet, quinine still as *certainly* annihilates malarial diseases, opium relieves *pain* and produces *sleep*, and mercury cures *inflammation* and *syphilis*. The art of medicine is based upon the accumulated facts and observations of ages. Its rules and principles are simply derived from these sources. Call it empiricism, or what you will, it is the nearest approach to truth bequeathed to us and can never be superseded by the Cook and Distiller of the theory of Alimentation and Stimulation. It is as irrational to declaim against the use of bleeding, mercury and tartar emetic because they are sometimes injudiciously employed, as it is to denounce food and wine and whisky, because their *abuse* leads to gout, intemperance, delirium tremens, paralysis and death.

The high claims set up by the friends of mercury, as to its beneficial effects in subduing inflammation and other diseases, and its adaptation especially to malarial fevers and affections of the liver, are disputed by its opponents. They deny it has any cholagogue action, most of them, and will not admit the theory of its absorption into the system. Todd, Bennett & Co., are noted for their opposition to calomel in these relations, and the boldness of their denial of its reputed virtues in inflammatory diseases and in acting upon the liver is very injurious to the cause of medical truth, especially in the case of many young and inexperienced members of the profession. Committees of prominent European medical men have been appointed to make special investigations of the action of these mercurial preparations upon the human system, and to inquire into the important question of their absorption. After elaborate inquiries and protracted labors directed to a knowledge of the action of mercury upon dogs and other inferior animals, and as far as possible upon man, embracing the feature of absorption, these gentlemen reported very unfavorably as to its action and value. It is even denied that it is absorbed into the human system, and the denial is willingly caught up by the opponents of calomel, and they are seeking to get up a greater hue and cry than ever before against it. Their experiments upon inferior animals are very fine illustrations of their devotion to physiology, and their fine spun theory as to the impossibility of introducing into the system by absorption, or any other possible way, globules or particles, however small or minutely reduced, of any preparation of mercury, reads well enough on paper, but unfortunately for their reasoning and conclusions, are overthrown by innumerable and overwhelming facts to the contrary. Let us apply the test of medical logic and of scientific induction to this question, and proceed to examine it fairly and clearly.

I have often seen under the operation of calomel almost apparently pure bile discharged by stool, and often have I seen bile discharged by vomiting in large quantities during the purgative action of the same mercurial; and how any one, conversant with this tendency, undisputed until recently, of calomel to increase the secretion of bile, I am utterly at a loss to conceive. How, if it is not absorbed, can you account for its action on the gums, or the dissolution of plastic deposits, its removal of serous effusions, its power of rapidly healing chancre, its unquestioned ability to cure iritis? How can we otherwise account for salivation producd by mercury introduced into the system by inunction or hypodermic injection? Let us summon to the stand, as a witness of great reliability and experience, the distinguished Headland, than whom there is no higher authority in our profession. In his admirable work "On the Action of Medicines on the System," this great light states that "*Mercury is absorbed into the blood, and particularly tends to the liver, bowels, salivary glands and skin.*" He classes it among the "true cholagogues." He says that "Mercurials increase more or less all the secretions, and even if we had no direct proof of their action on the liver, we might almost have affirmed that they especially increase the secretion of bile from the obvious way in which bilious symptoms yield to their action. But *we have direct proof of this.* M. Bucklein has made some careful experiments on a dog. Having given it mercury, he cut down upon the hepatic duct and collected the secretion and subsequently analyzed it. He found that the bile was increased and that mercury was found in it."—[Page 297.] Again he says: "Mercury, sulphur and iodine have been chemically detected in the perspiration. It has happened when a course of mercury has followed the administration of sulphur that parts of the skin have turned black from the formation of sulphuret of mercury."—[Page 301.] He also says that "mercury has been detected in the urine." —[Page 306.]

Let us now consult another learned and high authority, Pereira. He states, in his great work on Materia Medica and Therapeutics—[American Edition, 1852, vol. 1, page 773]—that, "By the external or internal use of mercury this metal becomes *absorbed*, and is subsequently either *deposited* in some of the *solids of the body*, or *thrown out* of the system by some of the *excreto-*

*ries.* The accuracy of the statement is proven by the following facts: 1st. Mercury has been detected in the blood by Zeller, Buckner, Schubarth, Colson and Dieterich. It appears to be in such intimate combination with the vital fluid that it cannot be recognized by the ordinary tests. *Destructive distillation* is in most cases necessary for its detection. 2d. Mercury has been found in the secretions, viz: in the perspiration, in the saliva, in the gastro-intestinal secretion, the bile, the urine, and in the fluid of ulcers. 3d. Mercury has been found in the organic solids, viz: in the bones, brain, synovial capsules, pleura, humours of the eye, cellular tissue, lunges, etc. In what part of the system reduction is effected has not been made out."—[Volume 1, pp. 773 and 774.]

"The secretion of bile (by mercury,) is promoted."—[Page 774.]

Who has not seen in the old treatment of remittent fevers, that just as soon as the gums were touched by mercury the liver poured out large quantities of blackish, green, viscid bile, which invariably denoted a favorable solution of the disease? But let us go on with the authorities. Our own illustrious American Professor, Wood, remarks as follows, in his able work on Therapeutics and Pharmacology—[2d vol., page 243]—"That mercury is absorbed is proved by the following facts: When rubbed upon the skin it in part disappears. After administration, it has been detected by chemical tests in the blood, saliva, perspiration, bile and urine, and is said to have been found in a metallic state in the brain, bones, cellular tissue, lungs, etc. Infants affected with syphilis are asserted to be treated effectually by the administration of mercurials to the nurse, etc. The hepatic secretion is often energetically stimulated, especially when the medicine is administered internally. There is no cholagogue which approaches in efficiency some of the preparations of mercury. A true cholera morbus, with copious vomiting and purging of bile, is not unfrequently induced by a large dose of calomel."

*Stille*, in his Therapeutics and Materia Medica (Vol. 2, p. 731), cites numerous and convincing proofs of the absorption of mercury. After mentioning the experiments of Schobarth and Zellar, before mentioned, he says: "Oesterlon found *minute globules of mercury* in the *pancreas, liver, lungs, heart, mesenteric glands, kidneys*, etc., and also in the *urine, bile, milk* and *saliva*," (p. 782). "Among the proofs of mercurial absorption by man, the following may be selected: In 1810 Brickmann published an account of a lady who, a year after being salivated, having become heated by violent dancing, *mercurial stains appeared* on her breast, and *metallic mercury* was found in her *linen*. In 1813 Jourda collected a quantity of mercury from the *urine* of a *syphilitic patient* who was *taking* this remedy." *Stille* quotes other authorities to similar facts, and treats at length, fully and clearly, of the effects of medicinal doses of mercurial remedies, their action—similar to that given by others—producing liquid and bilious discharges, etc." I might go on and cite additional authorities, numerous and high, in corroboration of the observations, experiments and statements of those I have given; but can any honest and unprejudiced man require further proof of the position that mercury is absorbed into the blood, and is a cholagogue?

The following additional quotations from Headland are well worthy of introduction here: "As hematic medicines mercurials have a double action. They counteract inflammation in general, and the poison of syphilis in particular, (p. 325). On account of the durable and effectual nature of its action, mercury is of great use in preventing the process of *effusion*, and in causing the *absorption of effused products*.. It is thus employed in *pleurisy*, and in other *membraneous inflammations*. Next to these it is most useful in inflammation of the *liver* and *brain*. It is inferior to antimony in fevers and rapid inflammations, because slower in operation, and without any direct action on the nervous system. In cases of primary syphilis mercury is *by far* the best medicine with which we are acquainted. It should be used in all cases, except where there is deep-rooted scrofula or marked debility, or a sloughing and irregular condition of the primary sore. It should always be given in Iritis."

"Mercury, being unnatural to the blood, passes at length out of the system through the glands, and acts as an eliminative. Like antimony, it tends to increase all the secretions in the body.

But whereas, antimony acts especially on the secretions of the skin and pulmonary membranes, mercury *tends particularly* to excite the *functions of the liver* and *bowels*, being *cathartic* and *cholagogue.*"

On another occasion Headland writes as follows: "Another remedy, of a different kind, has been used in all the diseases in which quinine is admissible, proving in some cases superior, and in other instances second only to it in its beneficial action. This is mercury; and in remittent and yellow fever; of the first importance in dysentery; employed by Dr. Baillie in ague, and pronounced by him to be superior in some cases even to quinia. In small doses it is frequently of use in cases of debility and scrofula. And mercury is a cholagogue, i. e., an agent which is known to have the effect of promoting the secretory function of the liver. Thus we may conceive that mercury, not given in excess, or to salivation, may operate in a different way to produce the same effect as quinine.

If the connection between tonics and the bile were actually established, then we should be enabled to explain a matter which otherwise would seem difficult to understand—how it is that small doses of mercury may sometimes act as tonics, though we know that the ultimate action of the medicine, like that of other catalytics, is to deterioate the blood. Even in scrofulous and enfeebled cases small doses of blue pills or calomel are often signally useful, and not prejudicial, as is sometimes stated by those who confound their application with that of mercury, given in salivating doses. Under such a course, when judiciously enforced, we may see the dilated pupil contract to its normal size, and the pale, enervated countenance become rosy and lively, and feel the weak and compressible pulse to become hard and firm. Perhaps mercury in such a case may be indirectly tonic, by restoring to the blood the natural tonic principle of the bile."

The reactions in the history of this valuable but much abused remedy of calomel, the fluctuations of professional appreciation of its importance and value, have been greater than attach to any other remedial agent of the whole profession. But when we remember that our best remedies are those which have been most abused, shall we reject the *good* which this powerful agent is capable of producing, because its *abuse* has been so great? To yield to the unfounded prejudices and noisy clamor against the use of calomel, which are becoming even traditional, that to use it in efficient doses places a practitioner in the ranks of old fogyism, and behind the rapid strides of medical science, is neither manly, philosophical, nor scientific. That physician is wanting in the stamina of real manhood, and in the sustaining power of true professional devotion, who allows any storms of surrounding circumstances, or any prejudice or tradition, however honored by time or authority, to stand between himself and a judicious trial of legitimate means, whether of ancient origin or modern growth, in combatting disease and saving life. I admit that much prudence and discrimination should be exercised in the administration of mercurials. I confess that I have often seen them used to such an excess in the bowel complaints of children, and other diseases of infancy and adult age, as to produce that deterioration of the system, that disintegration and disorganization of the blood, that entailed dropsy, consumption, and other protracted and wasting affections, which either greatly injured the constitution or ended in death. Such ignorance and mal-practice in the use of this effective weapon, while inexcusable, have contributed greatly to that discrepancy in the results of its administration which has made the calomel treatment objectionable to so many good men. But when we learn, as learn we must, more of its mode of action, as well as of its remarkable capabilities and most valuable adaptations in subduing many of the most fatal diseases of the human system, it is reasonable to suppose that less ignorance will guide, and less prejudice and danger attend its use. The authentic accounts from high authorities of the happy effects of large doses of a scruple and upwards of calomel in cholera morbus, epidemic dysentery, cholera, etc., are only equalled in striking import by the well-attested records of the cures of that terrible enemy of infantile life, membraneous croup, by similar doses, during the last twenty years, in the practice of most distinguished physicians of New York city, and elsewhere. The introduction of the calomel treatment in croup has been credited to Dr. Bay, of Albany, New

York; but Dr. Hamilton, in his work on mercury, published early in the present century, regards it simply as the American treatment, and says it spread from America to England. This celebrated author, who has written more strongly against the general use of calomel than almost any writer prior to those Modern Revolutionists, Todd, Bennett & Co., extolled its action in large doses nevertheless in genuine croup. He says that "no relief whatever has been afforded by that medicine (calomel), unless copious, dark-green colored stools, like boiled spinach, have been discharged, and that it requires large and repeated doses of the medicine to produce that effect. For example, to a child seven years old, one hundred and thirty-three grains were given within sixty hours." He says, moreover, that "in the only cases in which this medicine has failed under the author's direction (being in the proportion of *four* out of *fifty*), no evacuation through the bowels could be produced." "It is extremely difficult," remarks Professor Hamilton, "to explain, in the first place, the safety with which a hundred and thirty-three grains of calomel could be given in this climate (England) within sixty hours, to a child of seven years. Secondly, the relief which has invariablo followed the discharge of the dark-green colored evacuation."

Nowhere have I known or seen recorded such remarkable success in the treatment of croup, nor the necessity so strongly enforced of a pre-requisite to success of a continuance of the treatment until relief follows, or until there appears those free evacuations of the stools just described. I am told that scruple doses, and larger, are common in New York among the best practitioners, who treat croup successfully, although no published allusions are made to such treatment, owing to the prejudice against large doses of calomel, both within and without the profession.

The administration of heroic doses of this mineral under suitable cases and stages of disease received a revival in the example and endorsement of the illustrious James Johnson. He contracted a violent attack of dysentery while hunting on the banks of the Ganges. His medical attendants put him upon the usual treatment of small doses of calomel, combined with opium, together with mercurial inunction. This was continued for two days, but he constantly grew worse and worse. He says, in his own graphic account, that at this stage: "The Surgeon endeavored to cheer me, with the hope of ptyalism, which, he assured me would alleviate my sufferings. I then had no local experience in the complaint myself. As the night advanced, all the symptoms became aggravated, and I was convinced that a fatal termination must ensue unless a speedy relief could be procured. I had no other hope but on ptyalism, for my medical friend held out no other prospect. I sent for my assistant, and desired him to give me a scruple of calomel, which I instantly swallowed, and found that it produced no additional uneasiness; on the contrary, I fancied it rather lulled the tormina. But my sufferings were great—my debility was increasing rapidly, and I quite despaired of recovery! Indeed, I looked forward with impatience to a final relief! At four o'clock in the morning I repeated the dose of calomel, and at eight o'clock (or between sixty and seventy hours from the attack), I fell, for the first time, into a sound and refreshing sleep, which lasted till near midnight, when I awoke. It was some minutes before I could bring myself to a perfect recollection of my situation prior to this repose; but I feared it was still a dream, for I felt no pain whatever! My skin was covered with a warm moisture, and I lay for some considerable time without moving a voluntary muscle, doubtful whether my feelings and senses did not deceive me. I now felt an uneasiness in my bowels and a call to stool. Alas, thought I, my miseries are not yet over. I wrapped myself up to prevent a chill, and was most agreeably surprised to find that with little or no griping, I passed a copious, feculant, bilious stool, succeeded by such agreeable sensations—acquisition of strength, and elevation of spirits—that I ejaculated aloud the most sincere and heartfelt tribute of gratitude to heaven for my deliverance."

In addition to the slower and alterative action of calomel in minute doses, there seems to be a growing belief that when given in larger and purgative doses it is attended with *very decided sedative powers*. Dr. Leaming and others claim for it distinct sedative virtues, and the power to produce a profound and favorable impression through the sympathetic system of nerves upon those violent forms of disease common to Southern localities. But whether it acts mostly through absorption into the blood, or in no small degree, according to the theory of the reflex action enunci: ted by Marshall Hall, and which, through the investigations of Brown Sequard and others, have solved many intricate physiological and pathological problems, as many are assured, it cannot be denied, that, whether given in smaller or larger doses, as existing indications and changing circumstances should always decide, its use is indispensable in the treatment of a large number of the prevailing congestions, inflammations and other affections of this latitude.

Finally, in relation to the modern treatment of fever and inflammation, I have yet to learn of a single advocate of the depletory system who denies the influence of the conservative powers of nature in disease, or rejects the judicious administration of food and brandy. No considerate physician has ever denied that, when high fevers have been removed and inflammatory action subdued and other indications are favorable to alimentation, or when the system is much reduced either by acute affections, or slow, lingering diseases, suitable food and stimulation is not only proper but necessary. But that food and stimulants, except in limited quantities, are contra-indicated under opposite states and conditions, is a fact sustained by the general voice of the profession in this country and in Europe. The sensible physician never opposes, but always seeks, the aid of nature; the expectant system has its comparative value, and its remedies are sometimes appropriate and sufficient; and alimentation and stimulation have their subordinate and proper positions in the catalogue of remadial agents; but *singly*, or in *any combination*, such influences and agents, when brought to bear upon concentrated malaria, or directed against the higher grade of our congestive fevers and inflammations, are nothing more than mere "*meditations upon death.*" The disciples of a judicious antiphlogistic treatment are among the foremost in recognition of the influences named; they are ever ready and anxious to consult with nature, and to admit its full power in the class of self-limited diseases, and that under this self-limitation numerous cases will progress to recovery without medication. But all this is no evidence that, even in self-limited diseases, judicious treatment is not indicated—not alone to cut short, modify, and centrol disease, but also to prevent unnecessary complications, and sometimes to save life.

If the medical profession has no higher functions to perform than a mere skillful manipulation of the Liquor Distillery, as some have doubtless concluded who have read Dr. Norcom's address, it is a mere mockery to the community, and our science, instead of being humane and noble, as we have claimed it to be, is worthy of no higher devotion than such as the cook and distiller can bestow. If the views we have honestly sought to combat are correct, better, much better, would it be to disorganize all our medical organizations, break up our Medical Schools and Universities, and burn up our Medical Journals and Books. And then, in penitential sorrow for the offences of our past professional devotion, to come forward, in a spirit of humiliation and mortification, to admit, that the advancement of medicine and the reputed glory of our profession are elipsed by the triumphs of the kitcnen and the achievements of the distilleries and drinking saloons.

While, with good feelings towards Dr. Norcom, I am pleased to admire the ingenuity and ability of his address, yet I am induced to offer these opposing views and arguments from a sincere conviction that his doctrines are not in accordance with the teachings of medical truth, and of true medical progress; that they tend to throw discredit upon this Society, and upon the experienced practitioners of the State; that their tendencies are to bring the art and scienne of medicne into contempt with the ignorant and vulgar; and are pernicious in their influences upon the minds of the younger men of the profession.

He only is a true medical philosopher who alike interprets wisely the instructions of Nature and the indications of disease, and then pursues with patience and firmness the pathway illumined by their radient beams. Let them shun equally the dogmas of those extremists in medicine whose enthusiasm or prejudices are so liable to lead them from the true paths of legitimate practice. Pursuing the even tenor of his way, seeking only for truth, let him reject no doctrine because it is old, nor adopt any theory because it is arrayed in the captivating charms of novelty. Such a course may be slow to secure public attention and patronage, and may not strike the admiration of the fickle multitude; but in the end it will secure that kind of *permanent* success which no sneaking detraction or envenomed envy can shake, and which the superficial and wily medical demagogue never attains. His name will be enrolled high upon the list of noble and faithful physicians, and will go down as a rich and proud inheritance to his children and family. Such a conservative course, always to be united surely with personal integrity and honor, may not satisfy the ambitious aims of those who are not content with the *gradual* advancement of themselves, and the sure progress of a well-settled science. It may not serve to calm the restlessness of those who are ever on the alert, either to weave new theories themselves, or to adopt the new-fangled doctrines of others—sometimes for lack of judgment, but too often from a love of gain and notoriety. But, founded upon the rock of principle rather than expediency, he who takes this rough but alluring pathway, and pursues it with honesty and persistent industry, will surely reach that higher and more enduring basis of prosperity and renown that are the rewards of a high course, and which constitute the true ends of effort and the noble objects of life. These transcendental extremists may not do as much injury to society and medical science as those cunning, undermining, despicable, medical demagogues in our own regular ranks, who, under the authority of an ill-gotten diploma, smear their filthy slime on their daily ways of humbuggery and evil. Their motives are superior to those of these pests of the profession; but, whatever their purposes, they do more real harm to science and the profession than those irregulars who come out openly against us, and boldly pitch their tents outside the garrison of Legitimate Medicine.

